# Intolerance of Uncertainty and Emotion Regulation in Generalized Anxiety Disorder: The Role of Reappraisal and Suppression

**DOI:** 10.3390/bs15091238

**Published:** 2025-09-11

**Authors:** Letao Sun, Haochen Zou, Wei Li, Hui Li, Jiaoyan Pang, Huiru Cui, Chunbo Li

**Affiliations:** 1 Shanghai Mental Health Center, School of Medicine, Shanghai Jiao Tong University, Shanghai 200030, China; sd1f3117@126.com (L.S.);; 2 School of Psychology, Shanghai Jiao Tong University, Shanghai 200240, China; 3 School of Government, Shanghai University of Political Science and Law, Shanghai 200030, China; 4 Shanghai Key Laboratory of Psychotic Disorders, Shanghai 200030, China

**Keywords:** anxiety disorder, intolerance of uncertainty, emotion regulation, correlation analysis

## Abstract

To explore the correlation of intolerance of uncertainty (IU) and emotion regulation (ER) in patients with generalized anxiety disorder (GAD), a total of 108 GAD patients and 115 healthy participants were recruited. The intolerance of uncertainty scale (IUS) was used to evaluate the level of IU. The emotion regulation questionnaire (ERQ) was used to evaluate participants’ preference for emotion regulation strategies. The Hamilton anxiety scale (HAMA) and generalized anxiety disorder scale (GAD-7) were used to assess the degree of anxiety symptoms. Spearman correlation analysis and linear regression analysis were performed on participants’ scores. The scores of the IUS subscales, ERQ subscales, and HAMA in the GAD group were different from those in healthy controls (HC group) (*p* ≤ 0.001). IUS subscales were correlated with ERQ subscales, and inhibitory IU was significantly correlated with expression suppression strategies (*p* < 0.01). The level of IU is correlated with the selection of emotion regulation strategies in patients with GAD. Moderating effect analysis shows that emotion regulation strategies partially moderate the relationship between IU level and anxiety symptoms. This study underscores the critical role of intolerance of uncertainty and emotion regulation in shaping anxiety severity in GAD, highlighting the potential for targeted interventions that address both cognitive and emotional dimensions.

## 1. Introduction

Generalized anxiety disorder (GAD) is one of the most common mental disorders. The lifetime prevalence rate of GAD in the general population in China is 3.2% (95% CI 2.1~4.7%) (Huang et al., 2019). The symptoms are characterized by excessive and uncontrollable worry about a series of events or activities. Uncertainty is considered a core component of worry (Boswell et al., 2013). People with anxiety often show excessive worry about uncertain life events, react negatively in emotional and behavioral aspects, and develop symptoms of persistent stress. This phenomenon is known as intolerance of uncertainty (IU) (Shihata et al., 2016).

IU is considered as a risk factor for the development and maintenance of anxiety. People with elevated IU hold underlying negative core beliefs about uncertainty, have biased information processing in the context of ambiguity, and make threatening interpretations of uncertainty (Jacoby, 2020). As a trait associated with poor mental health, measuring IU accurately is vital for understanding a wide range of mental health conditions and for interventions (Lin et al., 2024). The most widely used tool for measuring levels of uncertainty is the Intolerance Uncertainty Scale (Buhr & Dugas, 2002). The scale consists of 12 items and has a stable two-factor structure. The two factors are prospective IU and inhibitory IU (Carleton et al., 2007). Prospective IU refers to goal-directed responses that affect cognitive expectations in the assessment of future events. The fear of an uncertain future event creates an association with feelings of anxiety, which activates maladaptive behavior (Maftei & Lãzãrescu, 2022). Inhibitory IU refers to the avoidance-directed response. The unknown anxiety affects the tendency of individuals to adopt avoidance behaviors to reduce exposure to uncertainty, thereby precluding the search for meaning in life events (Carleton et al., 2012).

Current research identifies IU as a critical factor in anxiety, exacerbating symptoms and influencing related domains. For instance, beliefs about emotion controllability may enhance interventions targeting IU and anxiety (Becerra et al., 2023), while anxiety sensitivity mediates the relationship between IU and somatic anxiety (Wright et al., 2016). As a transdiagnostic factor, IU underpins the similarity in repetitive cognitive activities between compulsive behaviors and anxiety symptoms (Gillett et al., 2018). These domains affect people’s emotion regulation, leading to worse feelings of anxiety. Compared to anxious people, GAD patients have more deficits in emotion regulation (Barlow et al., 2016). According to the emotion regulation model of GAD, GAD patients have a more intense emotion-generating process and are deficient in altering their emotional experience (Mennin et al., 2004). Therefore, appropriate emotional regulation helps to mitigate emotional distress (Fresco et al., 2013). According to the Process Model of Emotion Regulation proposed by Gross, people can use various strategies to regulate their emotions (Gross, 1998). Based on a factor analysis of this model, Gross divided emotion regulation strategies into two categories: cognitive reappraisal and expression inhibition (Gross & James, 1998). Cognitive reappraisal is a change made to an event before the emotional response begins. This strategy is a common and effective regulation strategy to reduce negative emotional experiences (Gross, 2015). Expression suppression is a reaction-centered emotion regulation strategy, which is embodied in the attempt to hide and change the original emotional response after the event (Simon et al., 2022). This strategy has been portrayed as a maladaptive method for managing emotion, deemed ineffective and effortful (English, 2024).

For GAD patients, the ability to regulate emotions effectively is more critical for psychological well-being, particularly in the face of uncertainty (Carleton et al., 2012). IU, as a trait characterized by excessive negative reactions to uncertain situations, may influence the preference for and effectiveness of these regulation strategies (Carleton et al., 2012). Clarifying the interplay between IU and ER can help uncover the mechanisms of anxiety and support the development of targeted interventions aimed at fostering adaptive coping. In this study, we first examined the correlations among IU, ER, and anxiety to clarify the direction and intensity of the linear association. Regression was then conducted to construct a model demonstrating the combined effect of IU and ER on anxiety. The subsequent mediation analysis explored the pathway through which IU influences anxiety by affecting ER. Overall, this study aims to explore the potential influence of IU and ER in the intervention of anxiety disorders, providing an objective basis for clinical tailoring intervention.

## 2. Materials and Methods

### 2.1. Participants and Ethics

This study was a cross-sectional study. A total of 108 GAD patients from a Shanghai mental health outpatient center and 115 healthy people with matched age, sex, and education level were enrolled from September 2022 to June 2024. Volunteers were invited to fill in an online form containing demographic data questions and self-assessment scales. The psychiatrist screened volunteers for mental health conditions using the Chinese version of the Mini-International Neuropsychiatric Interview (MINI). People who were screened without mental illness were invited to participate in the experiment as healthy volunteers. GAD patients were assigned into the GAD group, and healthy volunteers were in the healthy control (HC) group. The study was reviewed and approved by the Shanghai mental health center’s Institutional Ethics Committee with ethical approval code 2019-51C1. All participants signed informed consent. In this study, we did not use generative artificial intelligence (GenAI) to generate text, data, or graphics, or to assist in the study design, data collection, analysis, or interpretation.

GAD group inclusion criteria: ① meeting the diagnostic criteria for GAD in the International Classification of Diseases (ICD-10), and having the diagnosis reviewed by a psychiatrist using the Chinese version of the MINI. ② Hamilton Anxiety Scale (HAMA) score ≥ 14 points. ③ Hamilton Depression Scale 17-item version (HAMD-17) score ≤ 14 points. ④ Considering female menopause factors, the age range was selected as 18 to 45 years old. HC group inclusion criteria: ① no mental illness. ② Aged 18 to 45 years old. ③ Junior high school education experience or above. GAD group and HC group exclusion criteria: ① serious mental disorder other than GAD (such as schizophrenia or bipolar disorder), ② use of psychotropic substances, ③ serious physical disease, ④ suicide attempt, ⑤ pregnant or lactating women.

### 2.2. Assessment

In this study, IU levels and emotion regulation strategies were assessed through self-assessment. Anxiety symptoms were assessed by a psychiatrist and through self-assessment.

#### 2.2.1. Twelve-Item Intolerance of Uncertainty Scale (IUS-12)

The scale is rated on a Likert five-point scale, from 1 (“totally disagree”) to 5 (“totally agree”). A higher score means a higher level of IU. There was a 7-item subscale of prospective IU (IUS-P) and 5-item subscale of inhibitory IU (IUS-I). We used the Chinese version of IUS-12 and the internal consistency reliability of each dimension ranged from 0.704 to 0.878 (Zhang et al., 2017).

#### 2.2.2. Emotion Regulation Questionnaire (ERQ)

The ERQ is a 10-item scale developed by Gross and John in 2003 (Gross & John, 2003). There are 6 items on the cognitive reappraisal subscale (ERQ-C) and 4 items on the expression suppression subscale (ERQ-E). The scale is rated on a Likert seven-point scale, from 1 (“totally disagree”) to 5 (“totally agree”). The higher the score, the more frequently the emotion regulation strategy is used. Subjects with a higher frequency of cognitive reappraisal have better social ability and higher subjective well-being, while those with a higher frequency of expression suppression have the opposite effect (Zhao & Zhou, 2014). The retest reliability coefficients of the Chinese version of the scale are 0.85 and 0.87, and the criterion validity is above 0.70 (Wang et al., 2007).

#### 2.2.3. Hamilton Anxiety Scale (HAMA)

This scale is used to assess the severity of anxiety symptoms with 14 items. Each item is rated on a 5-point scale, ranging from 0 (not present) to 4 points (severe). The scale structure is divided into two major types of factors: somatic anxiety and psychic anxiety. A higher score means more serious anxiety symptoms (Hamilton, 1959). Three trained psychology researchers rated the participants on this scale.

#### 2.2.4. Generalized Anxiety Disorder 7-Item Scale (GAD-7)

The GAD-7 (Generalized Anxiety Disorder 7-item scale) is a self-report questionnaire used to screen for and assess the severity of GAD and other anxiety disorders. It consists of seven questions that ask about the frequency of specific anxiety symptoms over the past two weeks, using a 4-point Likert scale (0–3). Scores range from 0 to 21, with higher scores indicating greater anxiety severity (Spitzer et al., 2006).

#### 2.2.5. Hamilton Depression Scale (HAMD 17 Items)

This scale is used to assess the severity of depressive symptoms. There are 17 items, some items 0 (absent) to 4 (extremely severe). A higher score indicates more severe symptoms (Hamilton, 1960).

### 2.3. Analysis

We used IBM SPSS Statistics 27.0 (IBM Corp., Armonk, NY, USA) for statistical analysis. The Shapiro–Wilk method was used to test the normality of the data distribution in the GAD group and HC group. The test results showed that age, years of education, and HAMA scale were in line with a normal distribution. The IUS, ERQ, HAMD, GAD-7, and IUS subscale did not conform to normal distribution, so a Kruskal–Wallis test and Mann–Whitney U test were used for comparisons between groups. Furthermore, moderating analysis was used to examine the effect of emotion regulation strategies in the relationship between IU level and anxiety symptoms.

## 3. Results

### 3.1. Demographic Data

There was no significant difference in demographic data (age, sex, years of education) between the GAD group and the HC group (*p* > 0.05). Scores for the IUS subscale, ERQ-E, and HAMA in the GAD group were significantly higher than those in the HC group (*p* < 0.05); the ERQ-C score in the GAD group was significantly lower than that in the HC group (*p* < 0.05); and the HAMD score exhibited no statistical significance (*p* > 0.05) (Table 1).

### 3.2. Correlation Between IU and ER in GAD Patients

There were correlations between IUS subscales and ERQ subscales. IUS-P was negatively correlated with ERQ-C (*p* < 0.01), and positively correlated with ERQ-E (*p* < 0.05). IUS-I was negatively correlated with ERQ-C (*p* < 0.01) and positively correlated with ERQ-E (*p* < 0.01) (Table 2).

### 3.3. Correlation Between IU and ER in Healthy People

The HC group results showed that there was no statistical significance between IU level and the above indicators in healthy people (all *p* > 0.05).

### 3.4. Correlation Between IU and Anxiety Level in GAD Patients

IU was positively correlated with the HAMA somatic factor score (*p* < 0.01), HAMA (*p* < 0.05), total score of HAMA (*p* < 0.05), and GAD-7 (*p* < 0.05) (Table 3).

### 3.5. Correlation Between ER and Anxiety Level in GAD Patients

Cognitive reappraisal strategy (ERQ-C) was negatively correlated with HAMA somatic factor scores (*p* < 0.05), HAMA (*p* < 0.05), and total score of HAMA (*p* < 0.01). Expression suppression strategy (ERQ-E) was positively correlated with HAMA somatic factor score (*p* < 0.01), HAMA (*p* < 0.05), and total score of HAMA (*p* < 0.05) (Table 4).

### 3.6. IU, ER, and Anxiety Level Linear Regression Analysis in GAD Patients

The results of the correlation analysis revealed the influence of IU and ER on anxiety severity. IUS-P, IUS-I, ERQ-E, and ERQ-C were taken as independent variables, and HAMA was taken as the dependent variable for linear regression analysis. The results show that the model formula is as follows: HAMA = 6.41 + 0.28 × IUS-P + 0.31 × IUS-I + 0.14 × ERQ-E-0.12 × ERQ-C. The R^2^ of the model is 0.32 (Table 5).

### 3.7. Moderating Effect on IUS, ERQ-C, ERQ-E, and HAMA in GAD Patients

**Figure 1:**
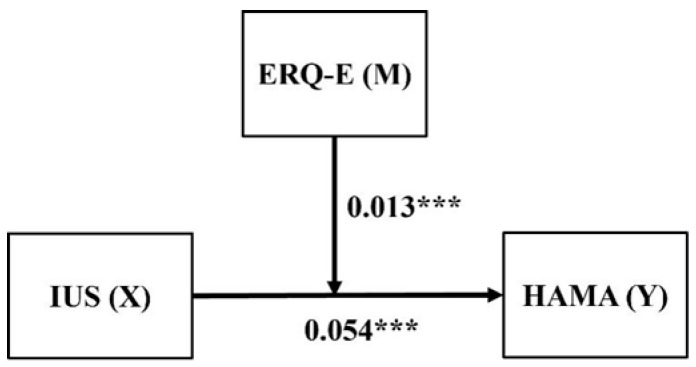
Results for moderating effect. (IUS: intolerance of uncertainty scale; ERQ-C: emotion regulation questionnaire—cognitive reappraisal; ERQ-E: emotion regulation questionnaire—expression suppression; HAMA: Hamilton anxiety scale. *** *p* < 0.001).

The moderating effect of emotion regulation strategies on the relationship between IU and HAMA was examined through the PROCESS program in SPSS. The results showed that ERQ-E had a significant moderating effect on the IUS-HAMA pathway (β = 0.013, 95% CI [0.006, 0.021]), while the moderating effect of ERQ-C did not reach statistical significance (β = −0.007, 95% CI [−0.022, 0.007]). The results showed that the relationship between IU level and anxiety symptoms can be regulated by expression inhibition strategies. A simple slope analysis indicated that among the participants who were used to expression inhibition strategies, the predictive effect of IUS on HAMA was the most significant (β = 0.424, *p* < 0.001), and this effect remained significant as this strategy increased (M + 1SD) (β = 0.566, *p* < 0.001). This result reflected the differentiated influence patterns of different regulatory strategies on psychosocial functions (Figure 1).

## 4. Discussion

The present study investigated the correlation between IU and ER strategies in patients with GAD, as well as their combined impact on anxiety symptom severity. These findings extend prior research by demonstrating that ER strategies partially moderate the relationship between IU and anxiety symptoms, highlighting the interplay between cognitive and emotional processes in GAD pathology. Previous research found that individuals with higher IU levels perceive uncertain situations as threatening, leading to heightened emotional reactivity and maladaptive coping responses (Boswell et al., 2013; Carleton et al., 2012). This is consistent with the present study. Notably, inhibitory IU was significantly correlated with expression suppression strategies, suggesting that GAD patients with elevated IU may employ avoidance-oriented regulation tactics to mitigate distress. This aligns with Gross’s Process Model of Emotion Regulation (Gross, 1998), wherein maladaptive strategies such as suppression fail to attenuate negative emotional arousal, instead exacerbating cognitive load and perpetuating anxiety (Compare et al., 2014). The moderating effect analysis further elucidated the role of ER strategies, revealing that ERQ-E significantly moderated the IU–anxiety relationship. This suggests that patients relying on suppression strategies exhibit stronger associations between IU and anxiety severity, perhaps due to the resource-intensive nature of suppression and its limited efficacy in altering emotional experiences (Gross & John, 2003). In contrast, ERQ-C did not demonstrate a significant moderating effect, possibly due to its adaptive nature, which may buffer the impact of IU on anxiety when employed effectively.

This study highlights the role of ER strategies in intervening with IU in clinical treatment. Effectively targeting and modulating the level of IU holds considerable significance for psychotherapy. Appropriate ER strategies may create the capacity to cope with what are perceived as catastrophic eventualities associated with uncertainty (Carleton, 2012). Based on our findings, treatment can emphasize the reappraisal of maladaptive cognition–emotion interactions. Stressful life events, such as neglect or trauma, frequently occur within unpredictable contexts, leading patients to adopt negative ER strategies in an effort to attain immediate certainty and perceived safety. These negative strategies often manifest as excessive attempts to control potential outcomes and hyper-vigilance toward environmental cues. Through guided cognitive reappraisal, patients can restructure their interpretations of uncertain situations. By engaging in self-observation and critically reflecting on cognitive biases, patients will learn to perceive uncertainties more objectively and recognize improper self-perceptions. The implementation of adaptive ER strategies facilitates a more interactive engagement with threat-related worries, thereby promoting symptomatic improvement and enhancing emotional well-being in patients with higher IU. The improvement in patients’ emotional regulation ability helps them learn to reframe uncertainty as a normal part of life rather than threatening, reducing catastrophic thinking and emotional reactivity. Relevant psychotherapy techniques may empower patients to respond to uncertainty with flexibility rather than fear, reducing anxiety and improving overall emotional well-being.

This study indicates that IU may be a necessary condition for the potential development of anxiety symptoms, which has certain implications for revealing that IU is a necessary trigger for anxiety symptoms. But there are still several limitations. First, its cross-sectional design precludes causal inferences regarding the relationships between IU, ER, and anxiety. Longitudinal studies are needed to explore how these factors evolve over time and interact during disease progression. Second, the sample was restricted to adults aged 18–45, limiting generalizability to other age groups. Future research should include broader demographics and explore comorbid conditions (e.g., depression) to clarify transdiagnostic mechanisms and investigate the efficacy of interventions combining uncertainty tolerance training with ER skill development, as well as their long-term outcomes in diverse clinical populations.

## 5. Conclusions

In summary, this study highlights the critical roles of IU and ER in GAD symptomatology, with expression suppression strategies exacerbating the adverse effects of IU on anxiety. Whether different ER strategies adopted by anxiety patients in the face of uncertain situations are related to broader emotional states, especially the interaction with other risk factors of anxiety disorders, will also be an important direction and content of future research.

## Figures and Tables

**Table 1:** Demographic and clinical data.

Index	GAD (n = 108)	HC (n = 115)	Statistics	*p* Value
Age/year	34 ± 7.6	33 ± 6.8	F = 0.53	0.983
Education/year	15 ± 2.4	17 ± 2.6	F = 0.96	0.265
Gender (male/female)/n	46/62	54/61	χ^2^ = 17.95	0.374
IUS-total/scale	31.91 (21–42) ^①^	20.63 (7–21) ^①^	χ^2^ = 16.32	<0.001
IUS-prospective/scale	17.76 (11–23) ^①^	12.87 (5–22) ^①^	χ^2^ = 18.13	<0.001
IUS-inhibitory/scale	14.15 (8–20) ^①^	7.76 (4–16) ^①^	χ^2^ = 30.42	<0.001
ERQ-cognitive reappraisal/scale	27.31 (13–38) ^②^	28.47 (16–38) ^①^	χ^2^ = 29.26	<0.001
ERQ-expression suppression/scale	15.92 (7–22) ^②^	11.78 (6–17) ^①^	χ^2^ = 39.64	<0.001
HAMA-somatic anxiety/score	8.2 ± 5.7 ^①^	2.1 (0–5) ^③^	χ^2^ = 43.52	<0.001
HAMA-psychic anxiety/score	5.9 ± 3.6 ^①^	1.3 (0–4) ^③^	χ^2^ = 38.94	<0.001
HAMA/scale	14.7 ± 8 ^①^	3 (1–6) ^③^	χ^2^ = 22.17	<0.001
HAMD/scale	5.8 (3–12) ^③^	2.7 (1–6) ^③^	F = 18.30	0.629

^①^ *p* < 0.001, ^②^ *p* < 0.05, ^③^ *p* > 0.05, GAD: general anxiety disorder, HC: healthy control, IUS: intolerance of uncertainty, ERQ: emotion regulation questionnaire, HAMA: Hamilton anxiety scale, HAMD: Hamilton depression scale.

**Table 2:** Correlation between IU and ER in GAD patients (n = 108).

	Mean	S.D.	IUS-P	IUS-I	ERQ-C	ERQ-E
IUS-P	17.76	6.57	1			
IUS-I	14.15	5.78	0.43 **	1		
ERQ-C	27.31	10.38	−0.26 **	−0.48 **	1	
ERQ-E	15.92	7.22	0.33 *	0.42 **	−0.42 **	1

Note. * *p* < 0.05, ** *p* < 0.01. IUS-P: intolerance of uncertainty—prospective. IUS-I: intolerance of uncertainty—inhibitory. ERQ-C: emotion regulation questionnaire—cognitive reappraisal. ERQ-E: emotion regulation questionnaire—expression suppression.

**Table 3:** Correlation between IU and anxiety level in GAD patients (n = 108).

	Mean	S.D.	IUS-Total	HAMA-S	HAMA-P	HAMA	GAD-7
IUS-total	31.91	10.46	1				
HAMA-S	8.22	5.74	0.38 **	1			
HAMA-P	5.91	3.60	0.33 *	0.30 **	1		
HAMA	14.69	7.99	0.35 *	0.84 **	0.88 **	1	
GAD-7	8.6	6.42	0.40 *	0.38	0.48 *	0.51	1

Note. * *p* < 0.05, ** *p* < 0.01. IUS-total: intolerance of uncertainty scale. HAMA-S: HAMA—somatic anxiety. HAMA-P: HAMA—psychic anxiety. HAMA: Hamilton anxiety scale. GAD-7: generalized anxiety disorder scale.

**Table 4:** Correlation between ER and anxiety level in GAD patients (n = 108).

	Mean	S.D.	ERQ-C	ERQ-E	HAMA-S	HAMA-P	HAMA	GAD-7
ERQ-C	27.31	10.38	1					
ERQ-E	15.92	7.22	−0.42 **	1				
HAMA-S	8.22	5.74	−0.29 *	0.32 **	1			
HAMA-P	5.91	3.60	−0.13 *	0.20 *	0.30 **	1		
HAMA	14.69	7.99	−0.45 **	0.26 *	0.84 **	0.88 **	1	
GAD-7	8.6	6.42	−0.28	0.31	0.38	0.48 *	0.51	1

Note. * *p* < 0.05 ** *p* < 0.01. ERQ-C: emotion regulation questionnaire—cognitive reappraisal. ERQ-E: emotion regulation questionnaire—expression suppression. HAMA-S: HAMA—somatic anxiety. HAMA-P: HAMA—psychic anxiety. HAMA: Hamilton anxiety scale. GAD-7: generalized anxiety disorder scale.

**Table 5:** IU, ER, and anxiety level linear regression analysis in GAD patients.

	*B*	*S.E*	*Beta*	*t*	*p*	VIF	Tolerance
Constant	6.41	3.12	-	2.06	0.041 *	-	-
IUS-P	0.28	0.08	0.23	3.35	0.001 ***	1.54	0.65
IUS-I	0.31	0.09	0.22	3.27	0.001 ***	1.46	0.69
ERQ-E	0.14	0.07	0.13	2.01	0.046 *	1.32	0.76
ERQ-C	−0.12	0.06	−0.16	−2.16	0.032 *	1.71	0.59
*R* ^2^	0.32						
*Adjusted R* ^2^	0.31						
*F*	*F* (4, 218) = 25.891, *p* = 0.000						
D-W	2.23						

Note: Dependent variable: HAMA. * *p* < 0.05, *** *p* < 0.001. IUS-P: intolerance of uncertainty—prospective. IUS-I: intolerance of uncertainty—inhibitory. ERQ-C: emotion regulation questionnaire—cognitive reappraisal. ERQ-E: emotion regulation questionnaire—expression suppression. HAMA: Hamilton anxiety scale.

## Data Availability

Data is unavailable due to institution ethical restrictions.

## Abbreviations

The following abbreviations are used in this manuscript:

GAD	General anxiety disorder
IU	Intolerance of uncertainty
ER	Emotion regulation
